# Evaluation of an international faculty development program for developing countries in Asia: the Seoul Intensive Course for Medical Educators

**DOI:** 10.1186/s12909-015-0518-8

**Published:** 2015-12-18

**Authors:** Do-Hwan Kim, Hyun Bae Yoon, Minsun Sung, Dong-Mi Yoo, Jinyoung Hwang, Eun Jung Kim, Seunghee Lee, Jwa-Seop Shin

**Affiliations:** 1 Department of Medical Education, Seoul National University College of Medicine, 103 Daehak-ro, Jongno-gu, Seoul 03080 Republic of Korea; 2 Global Center, Seoul National University College of Medicine, 71 Ihwajang-gil, Jongno-gu, Seoul 03087 Republic of Korea; 3 National Teacher Training Center for Health Personnel, Seoul National University College of Medicine, 71 Ihwajang-gil, Jongno-gu, Seoul 03087 Republic of Korea

**Keywords:** Collaboration, Faculty development, Southeast Asia, Kirkpatrick’s four-level training evaluation model

## Abstract

**Background:**

The issue of collaboration in medical education is becoming prominent. Some faculty development programs have suggested an approach for promoting collaboration on a global level. However, non-English-speaking developing countries in Asia, especially in Southeast Asia, do not take advantage of them due to their unique context, such as language and culture. To address these issues, Seoul National University College of Medicine initiated a 6-week international faculty development program called the “Seoul Intensive Course for Medical Educators” for 16 fellows from five Asian countries (Cambodia, Laos, Mongolia, Myanmar, and Vietnam). The aim of this study is to report the evaluation results of the outcome of the program and discuss better ways of collaborating with developing countries.

**Methods:**

Three levels of collaboration—intraorganizational, intranational, and international—were central initiatives of the program. Prior to setting up the program details, we first established four design principles, following which the contents, materials, and facilitators were determined to maintain consistency with the design principles. The evaluation of the program was done with Kirkpatrick’s four-level model. Most of the evaluation data for level 1 were collected by two questionnaires, the post-module survey and the post-program survey. Portfolios and progress reports were mainly used to collect outcome data for levels 2 and 3, respectively.

**Results:**

The reaction was generally positive throughout the program and there was a significant correlation between satisfaction and relevance to one’s job or needs. Despite the fellows’ propensity for overestimating themselves, both the evaluators and fellows reported that there was significant improvement in learning. Opinions on the impact or urgency of the topics were slightly different from country to country; however, the answers regarding feasibility were fairly similar. Moreover, we could observe from the post-program progress reports that the transfer of learning was actively in progress, mainly for topics that were highly feasible.

**Conclusions:**

These results show that the program was successful in terms of its effectiveness. Consistent and timely support is essential for the sustainable development of the medical education systems in these countries. Further understanding of the underlying factors on transfer (level 3) could improve the effectiveness of faculty development programs for developing countries.

## Background

The issue of collaboration in medical education is becoming prominent [1]. The spectrum of collaboration is broadening from the individual level, such as interprofessional education, to the institutional level and even further to the transnational level [2]. The education of health professionals including faculty development is no exception to this trend. Some programs, like the education programs of the FAIMER Institute [3] and Global Health Sciences [4], are worth mentioning as examples of international collaboration, which have already begun to show their effectiveness [5, 6]. What makes these programs more meaningful is that they emphasize building partnerships with developing countries. For countries where there are limited resources and financial constraints, collaboration among academic institutions is particularly important [7].

Despite growing trends for international collaboration, developing countries are still experiencing hardships, not only in recruiting human resources for medical schools but also in making opportunities for academic education [8]. Their efforts to expand human resources that are capable are frequently obstructed by a lack of a well-trained faculty who can provide professional training in the field of public health education, including medical education [9]. From a global perspective, addressing these problems is critical and listed in the WHO’s agenda for Post-2015, which targets achieving quality healthcare administered by human resources [10]. Because medical education changes the practice pattern, it can eventually have wide-ranging effects on the health of a population, especially in underdeveloped regions [11]. Thus, appropriate faculty development programs (FDPs) based on the contexts and needs of developing countries are necessary as a means to overcome these challenges.

When focusing on a Southeast Asian context, there are region-specific issues to consider. First, Southeast Asia is one of the regions with the smallest number of medical schools per million population [11]. The result of this shortage is an insufficient number of physicians and a lack of human and institutional capacity to address the healthcare problems of the people in this region [12]. Therefore, it can be stated that medical education in Southeast Asia is not properly responding to the needs of the region [13]. Second, the medical education culture in Asia is fairly different from that of Western countries, and even in Asia, the cultural and community needs differ among subregions [14]. These diverse cultures may not match with other cultural contexts, especially those of Western countries. Third, although there are international FDPs, provided by developed countries for academic institutions in developing countries, most of the countries in Southeast Asia have hardly gained any benefits from these programs. In addition to all sorts of costs coming from participating in programs that are held in geographically distant countries for any period of time, the biggest barrier is language, e.g., English language ability. For example, the FAIMER Institute requires their applicants to have a high level of proficiency in English [15]. For people born and raised in non-English-speaking countries, a lack of English ability is one of the most common obstacles blocking effective international collaboration [16]. According to the global metrics on brain drain, not speaking English seems to have a protective effect against brain drain [17], but ironically, it has also become a barrier for ‘brain-gain’ currently. Fourth, some countries in Southeast Asia have tried to run their own institutions for faculty development [18] but a lack of funding and experience are major constraints in achieving self-sufficiency.

After recognizing these challenges and disadvantages that non-English-speaking Asian developing countries face, Seoul National University College of Medicine (SNUMC) initiated an international FDP called the Seoul Intensive Course for Medical Educators (SICME). The Department of Medical Education (DME) and two affiliated institutions of SNUMC, the JW Lee Center for Global Medicine (CGM) and the National Teacher Training Center for Health Personnel (NTTC), had pivotal roles in planning and implementing the program. The primary and immediate goal of the SICME was to educate and prepare fellows as medical educators who would have a broad and general competency in the field. In the long term, we expect them to form a critical mass of leaders in medical education, and to drive educational reform in their own institutions and countries.

In total, 16 fellows from five Asian developing countries were invited to SNUMC for the program. Four countries—Cambodia, Laos, Myanmar, and Vietnam—belonged to Southeast Asia and one country, Mongolia, to East Asia. Mongolia was the only country located in a different geographic region, but WHO classified it in the Western Pacific Region, together with Cambodia, Laos, and Vietnam. Indeed, they share common problems in human resources for healthcare [18] as well as experience similar situations as non-English-speaking developing countries in Asia.

This study has two aims. The first one is to describe the key features of the program and to examine the evaluation results based on Kirkpatrick’s four-level training evaluation model to understand the effects and limitations of the program. The second aim is to discuss better ways to collaborate with developing countries in faculty development and education to overcome the challenges facing them. Even though the participants of the SICME are mainly from Southeast Asian countries, we also discuss the relevant implications that would be applicable to other non-English-speaking developing countries, especially those countries who have been marginalized from existing international collaboration due to language barriers.

## Methods

### Collaboration as the initiative of the program

Three levels of collaboration were central initiatives of the program. One level was intraorganizational collaboration inside the host institution—DME, CGM, and NTTC—as mentioned above. Another level included two types of intranational collaboration—one among fellows from the same countries and the other among facilitators from various medical schools in South Korea. The last one was at the international level, which included collaboration among fellows from different countries and continuing international collaboration among institutions participating in the SICME.

### Four design principles

Prior to determining the detailed curriculum of SICME, the Planning Committee, consisting of ten faculty members from eight medical schools, reached a consensus on four overarching design principles of the program.

1. To promote the fellows’ ownership of the program, every component of the SICME must be organized based on the careful consideration of the needs of the fellows, their institutions, and their countries.
2. To enhance the application and transfer of learning after the program, the contents must be adaptable in resource-limited settings. Additionally, every piece of educational material should be provided in both hard copy and soft copy formats, unless there are any copyright issues.
3. To facilitate learning, participatory and learner-centered pedagogical methods rather than teacher-centered ones must be prioritized and maximized throughout all courses.
4. For continuous improvement, evaluation of the program must be conducted based on Kirkpatrick’s four-level model and feedback from all participants must be considered.

### Module topics and facilitators

Another major task of the Planning Committee was to determine the topics for the 6 week program. Because the NTTC possesses its own source of education programs for health professionals which have been developed, revised, and used for over 35 years since its inception, module topics and their contents and relative lengths were determined primarily based on the previous education experience of the NTTC. After developing the broad outlines of the program, the Planning Committee thoroughly examined the educational backgrounds, professional experiences, stated needs and interests of each participant in his/her application form for fine adjustments to the program contents. All the members of the Planning Committee had iterative meetings to reach a consensus on the final structure and detailed contents of the program. Finally, one week was allotted for modules 1, 2, 3, 4, and 5. Modules 6, 7, and 8 were given one-third each of week 6 (Table 1). The whole program mainly comprised general topics on various areas of medical education, but topics such as organizational management and leadership were also included, taking into account the current positions and expected roles of the fellows after completing the SICME.

**Table 1:** Overview of the SICME

Week	Modules and topics	Total hours of education per module	Number of facilitators involved
1st week	Module 1: Theory & practice of teaching and learning	27	5
2nd week	Module 2: Curriculum development and evaluation	27	7
3rd week	Module 3: Student assessment	27	6
4th week	Module 4: Technology in medical education	15^a^	4
5th week	Module 5: Educational administration	27	6
6th week	Module 6: Student selection and admissions	9	2
	Module 7: Accreditation and licensing examination	9	3
	Module 8: Official development assistance for human resources development	9	1
Total		150	23 ^b^

^a^Two days of the New Year’s Holiday were in the 4th week; ^b^ Seven facilitators participated in more than two modules

After finalizing the whole layout of the program, members of the Planning Committee were divided among the modules and became Module Coordinators. The main role of the Module Coordinators was to establish a detailed teaching plan that was in agreement with the three major design principles. They took charge of some sessions in the module, but also recruited facilitators from other institutions when necessary. In addition to the 10 Planning Committee members, 11 faculty members from 14 medical schools and two experts on organizational development participated in the SICME as facilitators, making a total of 23 facilitators (Table 1). The teaching members were called ‘facilitators’, following design principle 3, which indicated our intention to facilitate learning for the fellows through student-centered rather than teacher-centered learning during the modules.

### Selection of the fellows

The SICME was held from January 6 to February 14, 2014. The total length of the program and the specific dates of the program were determined based primarily on the availability of various resources of the host institution, such as adequate space for the sessions, the availability of the facilitators, accommodations, academic calendar and the schedules of the students.

Shortly after determining the date, to invite the fellows, we sent an official letter which contained an overview of the SICME including its background, objectives, and schedule to the partner institutions of the five countries, and requested the recommendation of eligible candidates. After thoroughly reviewing the backgrounds, relevant experiences, and letters of recommendations of the applicants, the Selection Committee confirmed the final list of 16 fellows. We deliberately selected fellows who were in high positions in their organizations, such as a vice-rector, team leader, vice-dean, and education director of a development center at a university. There were two main reasons for selecting fellows based on these criteria. First, because the aim of SICME was to drive educational reform at the institutions of the fellows to bring about meaningful institutional change, we regarded change in the leaders themselves and in the leadership to be essential. The other reason was to foster a regional network of high-level administrators with the expectation for that to evolve into a continuing, cooperative relationship even after the program.

### Program materials and support

All the lectures and workshops were basically given in English, and education materials were also prepared in English because it was the only common language among the participants including the facilitators. One or more teaching assistants attended every session to assist the facilitator in conducting the workshops or in leading various kinds of activities. One laptop per fellow was provided so that they could easily search dictionaries or access the internet as needed. There was also one support manager who took care of the daily lives of the fellows outside of the classroom. Accommodations and per diem were provided by the CGM during their stay in South Korea.

### Evaluation

In this study, we used Kirkpatrick’s four-level model [19], which has been widely suggested for the evaluation of FDPs [20, 21]. For the level 1 evaluation, we conducted two surveys. First, a ‘post-module survey (PMS)’ was done immediately after finishing each module. Here, we mainly asked whether the fellows were satisfied with the various aspects of a module, and a four-level Likert scale was used from 1 (very unsatisfied) to 4 (very satisfied). Second, we conducted a ‘post-program survey (PPS)’ immediately after finishing the whole 6-week program. In the PPS, we asked the fellows to rate their overall satisfaction with the SICME (PP1) and to compare the relative merits of the 8 modules (PP2—PP5). For PP1, we increased the Likert scale to six levels from 1 (Very unsatisfied) to 6 (Very satisfied) because in the PPS, most of the answers were concentrated primarily on 3 (satisfied) or 4(very satisfied). All questionnaires were written in English, and we encouraged the fellows to ask the teaching assistant or their colleagues for help if they had any difficulty in understanding and answering the questions. Although both the PMS and PPS were not mandatory, all 16 fellows participated in the surveys after every module and after the whole program.

Two methods were used for the level 2 evaluation. First, four independent evaluators, professionals in medical education evaluated the portfolios that the fellows submitted before and after each module. The portfolios were anonymized by removing the name and nationality of the fellow before they were given to the evaluators. After reviewing each portfolio, the evaluators simply rated the overall competency of each fellow between 1 (Novice/Knows what) and 5 (Expert/Mastery) referring to two widely used competency level frameworks suggested by Dreyfus H [22] and Miller GE [23]. In addition, they also evaluated whether a fellow, who had completed the program, became suitable as an independent workshop facilitator. Second, we asked fellows to self-assess their learning after each module and after the whole program. While answering the PMS, fellows simultaneously self-assessed their overall competency using the same five-level competency scale that the evaluators used. Additionally, in the PPS, they were asked to assess their capability of running a workshop independently for each module topic (PP 6).

Because it is recommended to do the level 3 evaluation 3 to 6 months after training, we requested that the fellows report their actual progress 3–4 months after they completed the SICME. Moreover, we had designed part of the PPS with the purpose to predict the transferability of their learning by asking about the anticipated impact (PP 7), urgency (PP 8), and feasibility (PP 9) of the topics in the 8 modules.

To ensure content validity, all the instruments for data collection were designed and developed with reference to the book by Kirkpatrick DL [19], the designer of the model we used. During the process, the Planning Committee reviewed all the methods and materials used in the evaluation and when there was any disagreement among the members, it was discussed until a consensus was reached. All statistical analyses were conducted by IBM SPSS Statistics for Windows Version 20 (IBM Corp., Armonk, NY, USA). Independent sample *t*-test and Spearman’s correlation analysis were used to analyze the quantitative data obtained during the evaluation process.

### Ethical approval

For this study, exemption from ethical review was approved by the Institutional Review Board (IRB) of Seoul National University College of Medicine. Before conducting the surveys, we obtained oral informed consent from all the fellows, and the IRB waived the documentation of informed consent later in the ethical review process.

## Results

### Level 1. Reaction

Table 2 shows the results of the PMS and PPS questionnaires. For the PMS, the values for overall satisfaction for each module ranged from 3.44 to 3.73, and the mean value for the overall satisfaction for the whole program was 3.57 ± 0.50 on a 4-point Likert scale (Table 2). For the PPS, every fellow answered 5 or 6 on a 6-point Likert scale, and the mean value for the overall satisfaction for the whole program was 5.47 ± 0.52 (Table 3).

**Table 2:** Evaluation of level 1 (reaction) of the Kirkpatrick model (Post-module survey, *n* = 16)

Questions	Level of satisfaction (average of 8 modules, Mean ± SD)	Spearman’s rho^b^
PM 1. Contents^a^	3.68 ± 0.47	0.555
PM 2. Facilitators^a^	3.72 ± 0.45	0.703
PM 3. Facilities^a^	3.63 ± 0.48	0.810
PM 4. Materials^a^	3.63 ± 0.48	0.390
PM 5. Schedule^a^	3.51 ± 0.53	0.542
PM 6. Relevance to one’s needs and interests^a^	3.71 ± 0.46	0.454
PM 7. Proportion of activities to lecture^a^	3.52 ± 0.52	0.476
PM 8. Achievement of stated goals and objectives^a^	3.55 ± 0.52	0.571
PM 9. Help to do one’s job better^a^	3.66 ± 0.48	0.915^*^
PM 10. Overall satisfaction^a^	3.57 ± 0.50	-

^*^p < 0.01

^a^Rating scale: 1 (Very poor) – 4 (Very good); ^b^correlation between the rank of each module in item PM 10 (Overall satisfaction) and the rank of each module in the rest items (PM 1 – PM 9, PP 1)

**Table 3:** Evaluation of level 1 (reaction) of the Kirkpatrick model – comparison between modules (Post-program survey, *n* = 16)

Questions	Module 1	Module 2	Module 3	Module 4	Module 5	Module 6	Module 7	Module 8	Spearman’s rho^c^
PP 1. Overall satisfaction^a^	5.47 ± 0.52								-
PP 2. Most Relevant to one’s personal needs^b^	10	8	9	6	2	2	3	8	.890^*^
PP 3. Most helpful^b^	6	6	6	8	7	1	5	9	0.457
PP 4. Hopes to be lengthened^b^	0	2	4	4	3	6	7	15	−0.267
PP 5. Hopes to be shortened^b^	2	0	1	2	4	1	2	1	−0.329

^*^p < 0.01

^a^Overall satisfaction with the SICME program as a whole. Rating scale: 1 (Very unsatisfied) – 6 (Very satisfied); ^b^Fellow were asked to select three or less modules per item. The number of fellows who selected the modules is shown in the cells – i.e. maximum is 16 and minimum is 0; ^c^correlation to the item PM 10 (Overall satisfaction for each module)

Two items, one from the PMS and the other from the PPS, showed statistically significant correlations with the level of satisfaction for each module. One was ‘PM 9’ which asked how much a fellow thinks the module will help him/her do his/her job better (Table 2), and the other was ‘PP 2’ that asked fellows to choose three or less modules that were the most relevant to their own needs (Table 3).

### Level 2. Learning

In the PMS, we asked the fellows to self-assess their improvement in knowledge, skills, and attitudes. One noticeable finding was that they assessed their improvement in attitude higher than that in knowledge or skills. Although the difference was not statistically significant, the trend was consistent throughout the program, except for module eight (Table 4). This result is also consistent with the remarks of a facilitator who participated in four modules over 4 weeks—weeks 1, 3, 5, and 6.

**Table 4:** Evaluation of level 2 (learning) of the Kirkpatrick model: self-assessment in post-module survey (*n* = 16)

Questions	Module 1 (Mean ± SD)	Module 2 (Mean ± SD)	Module 3 (Mean ± SD)	Module 4 (Mean ± SD)	Module 5 (Mean ± SD)	Module 6 (Mean ± SD)	Module 7 (Mean ± SD)	Module 8 (Mean ± SD)	Average^b^
PM11. Improvement of knowledge^a^	3.44 ± 0.51	3.38 ± 0.50	3.53 ± 0.52	3.40 ± 0.51	3.63 ± 0.50	3.31 ± 0.48	3.31 ± 0.48	3.63 ± 0.50	3.45 ± 0.50
PM12. Improvement of skills^a^	3.25 ± 0.45	3.25 ± 0.45	3.40 ± 0.51	3.33 ± 0.49	3.38 ± 0.50	3.25 ± 0.45	3.25 ± 0.45	3.38 ± 0.50	3.31 ± 0.46
PM13. Improvement of t attitudes^a^	3.50 ± 0.52	3.44 ± 0.51	3.73 ± 0.46	3.53 ± 0.52	3.69 ± 0.48	3.44 ± 0.51	3.44 ± 0.51	3.50 ± 0.52	3.53 ± 0.50

^a^Rating scale: 1 (not improved at all)—4 (very much improved); ^b^the average for all eight modules

*“It is not easy to translate my impression into a quantitative measure. However, what I’ve found out during the SICME is that their (fellows’) way of thinking changed substantially and was constantly maturing as the SICME progressed. At week 6, the level of discussion and their attitudes as a medical educator were totally different from what I saw in the first week.”*

**Fig. 1:**
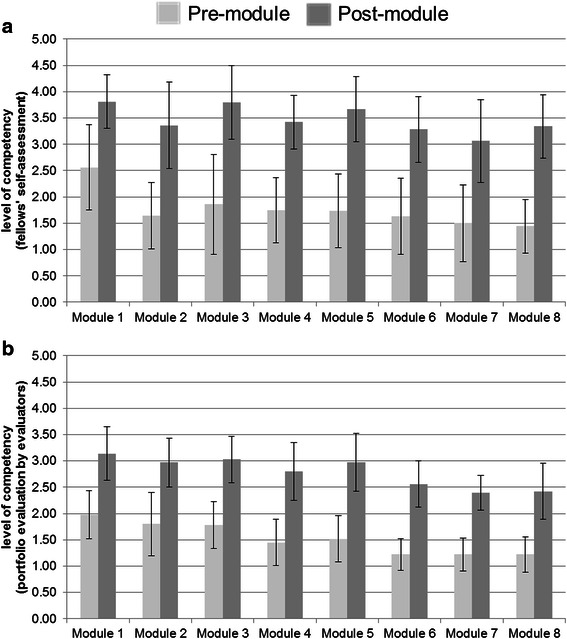
Evaluation of overall competency of the fellows for each module. Results of self-assessment by the fellows (**a**) and portfolio assessment by the evaluators (**b**) showed significant improvement in the post-module competency for all modules, compared to the pre-module competency (*p* < 0.001). The results of the self-assessed post-module competencies for each module, except for module 2, were significantly higher than that of the portfolio evaluations (*p* < 0.01), whereas the self-assessed pre-module competencies were higher only in module 1 (*p* = 0.019) and module 6 (*p* = 0.045). Error bars indicate standard deviations. Rating Scale: 1 (Novice/Knows what), 2 (Advanced beginner/Knows how), 3 (Competent/Shows how), 4 (Proficient/Does), and 5 (Expert/Mastery)

To evaluate learning, four evaluators independently assessed the portfolios of the fellows, and the fellows also self-assessed their own overall competencies (Fig. 1). Statistically significant improvement was observed in both results, but the results of the self-assessment were generally higher than the portfolio evaluation results for both pre-module and post-module competency.

Because one of our major goals was to train trainers who can then facilitate educational reform at their own institutions, the fellows were expected to disseminate what they learned during the SICME after going back to their countries. Therefore, not only improvement in knowledge, skills, and attitudes but also the development of actual capability as a workshop facilitator who can train trainers is important. In this regard, in PP 6, we asked fellows to choose all the module topics for which they could run a workshop on by themselves after going back to their home institutions. Meanwhile, evaluators also evaluated the suitability of each fellow as a workshop facilitator based on his/her portfolio. The results from the fellows and the evaluators were in agreement in that the fellows are the most capable for facilitating workshops for modules 1 and 3, and the least capable for modules 7 and 8. The correlation between the evaluation results of the evaluators and the fellows also showed statistical significance (Table 5).

**Table 5:** Evaluation of level 2 (learning) of the Kirkpatrick model: fellows who can run a workshop by themselves

	Module 1	Module 2	Module 3	Module 4	Module 5	Module 6	Module 7	Module 8	Average^c^ (Mean ± SD)	Spearman’s rho^d^ (*p*-value)
Fellows (Self-assessment)	16^a^	12^a^	15^a^	7^a^	5^a^	6^a^	3^a^	3^a^	8.38 ± 5.23	0.747 (0.033)
Evaluators	11^b^	8^b^	13^b^	9^b^	10^b^	9^b^	4^b^	5^b^	8.63 ± 2.97	

^a^the number of fellows who assessed themselves to be competent to run a workshop on the module by themselves in PP 6—i.e. maximum is 16 and minimum is 0; ^b^the number of fellows who were assessed, by one or more evaluators, to be competent enough to run a workshop on the module by themselves—i.e. maximum is 16 and minimum is 0; ^c^the average for all eight modules; ^d^correlation between the results from the fellows and the evaluators

### Level 3. Behavior/transfer

**Fig. 2:**
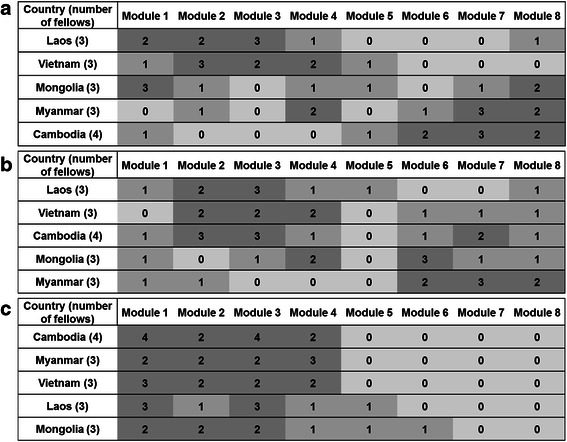
Comparison of impact, feasibility and urgency among module topics. Fellows were asked to select three modules per question, each of which asks about the impact (**a**), urgency (**b**), and feasibility (**c**) of the application. The brightness of a cell represents the number of fellows who selected the module—darkest (≥ two fellows), middle (one fellow), lightest (none of the fellows)

Prior to collecting actual data on transfer (level 3), we tried to examine how fellows perceived each module topic in terms of its impact, urgency and feasibility in the PPS (Fig. 2). As the topics with the most impact, the Laotian and Vietnamese fellows ranked modules 1, 2, 3 and 4 higher, while the Myanma and Cambodian fellows answered that modules 7 and 8 would have more impact. For the most urgent topic, the Laotian, Vietnamese and Cambodian fellows judged modules 2 and 3 to be more urgent. The Myanma fellows described modules 6, 7, and 8 as urgent. For the most feasible topics, fellows mainly chose modules 1, 2, 3, and 4, but some the Laotian and Mongolian fellows thought module 5 was also feasible.

The interim progress report for each country shows that fellows were actively sharing and transferring their experience from the SICME (Table 6). There were three countries for which projects on modules 1 and 2 were in progress, and a project on module 3 was on the verge of being implemented in Vietnam. In the two countries for which module 5 was seen as feasible, projects targeting organizational capacity building were, in fact, in progress. This contrasts the three other countries—Cambodia, Myanmar, and Vietnam—where the projects mostly targeted improving the capacity of individuals.

**Table 6:** Evaluation of level 3 (transfer) of the Kirkpatrick model: summaries of the progress report from five countries

Country	Title of the project	Target participants	Related SICME Modules
Cambodia	Workshop on Lesson Plan	33 faculty members of UHS	Module 1, Module 2
Laos	– Project 1. Provincial TOT Workshop	– Project 1: Training Committees from 5 provinces of Lao PDR, in total, 60 participants.	Module 1, Module 2, Module 5
	– Project 2. Asian Academic Partnership Consortium	– Project 2: 40 participants from University of Health Sciences, Faculty of Basic Sciences, Faculty of Medicine and Faculty of Postgraduate Study. 5 from Patan Academy of Health Sciences, Nepal.	
	– Project 3. Training Management Workshop	– Project 3: Training Management Committees from 5 provinces of Lao PDR, in total, 36 participants.	
Mongolia	Establishing Faculty Development Center	Private and Public University Faculties	Module 5
Myanmar	– Project 1. Curriculum development in line with accreditation	– Project 1: Members of the Academic board who are involved in curriculum development will be invited to Professor Shin's workshop	Module 1, Module 2
	– Project 2. Evaluation of teaching learning aspects using a Teaching Perspective Inventory (TPI) survey in basic science teaching faculty	– Project 2: Teaching faculty of basic science subjects at the University of Medicine 1	
	– Project 3. Medical education introduction for Junior teaching faculty	– Project 3: Junior teaching faculty	
Vietnam	Student Assessment Development	New medical educators who have experiences less than 2 years.	Module 3

Not long after the submission of the progress report, three of the five countries—Laos, Mongolia and Myanmar—requested that SNUMC provide further support and consultation as an external expert body. Hence, SNUMC organized three separate teams according to the topics and types of projects for which they requested support. Faculty members of SNUMC who participated in the SICME as facilitators took charge of each team as core members. In Myanmar, DME and CGM provided a three-day workshop in July 2014 on ‘Curriculum Development in Line with Accreditation’ to heads, directors, administrators from key organizations in Myanmar who have a major influence on medical education in that country including major medical schools, Myanmar Medical Council, and Myanmar Medical Association. In Laos, CGM, in collaboration with the Western Pacific Regional Office of WHO and University of the Philippines Manila, provided three one-week workshops that cover general topics of medical education to faculty members of the University of Health Sciences, the only medical school in Laos. Lastly, to support the Mongolian fellows, DME acquired funding for a three-year project from the Korea International Cooperation Agency (KOICA) which aims to strengthen the educational capacity of Mongolian health professionals. For this project, DME organized a joint team with experts in organization development who joined the SICME as facilitators in Module 5 and with Raphael International, a medical nonprofit organization whose primary activity is to provide medical aid to impoverished people, which has more than 8 years of experience in medical service provision in Mongolia.

## Discussion

In this study, we organized an international FDP for Asian developing countries, based on four design principles using multi-level collaboration. The results show that it is was successfully implemented with fellows who had limited training opportunities to address their own needs. The training was also effective in terms of the first (reaction), second (learning) and third (transfer) levels of Kirkpatrick’s model. Despite the effectiveness of the program, further studies are needed on each level and on how the collaboration actually worked.

First, for level 1 (reaction), the fellows showed generally high levels of satisfaction for the various aspects of the program. Although high satisfaction itself does not guarantee learning, this is important because participation might decline unless the fellows perceive that the time and effort they invested is meaningful. Thus, as we expected from our first design principle, it is important to consider the personal and professional needs of participants to motivate their participation [24, 25]. Actually, based on our data, a correlation between satisfaction and a module (PM 10) and the module’s relevance to the fellows’ personal needs and interests (PP 2) was statistically significant (Table 3), although those two were collected independently at separate time points—the former at each PMS and latter at the PPS. The perception of the benefit from a module (PP 3) or appropriateness of the allocated time to a topic (PP 4, PP 5) did not show any significant correlation to the satisfaction. Here, we could infer that careful examination of the needs and interests of the trainees is an effective way to achieve a high level of satisfaction as well to develop international FDPs for developing countries.

Regarding level 2 learning, one of the most interest findings was that a larger increase in attitude, which is particularly important in adult learning, was more significant than that of knowledge or skills. The third design principle of the SICME, which emphasized facilitating learning through learner-centered teaching methods and teacher-centered lectures, seems to have contributed to this result. Based on this principle, we reduced the proportion of lectures, to as low as 30% and tried to maximize learner-centered methodologies, such as small- and large-group discussions, role-playing, and participatory workshops. Through those learner-centered activities, what we tried to achieve was to provide fellows from five different countries with the opportunity to rethink their attitude as medical educators while encountering the ideals and reality of other countries, finding similarities and differences with each other, and arguing for and against something. Through these experiences, they exhaustively reflected on themselves and their situations and thought about their future direction. Previous research showing that active learning and student-centered approaches foster positive attitudes towards the subject [26] further supports this result.

Although the portfolio evaluation results showed significant improvement in the overall competency of the fellows, we found a tendency for the fellows to overestimate their own competency in the self-assessment results. This is not unusual because prior research has shown that doctors have a weakness in evaluating their own abilities [27]. However, there are also some positive aspects of this overestimation because high self-efficacy is one of the main learner characteristics necessary to bring about successful transfer, together with motivation and perceived utility [28].

Learning in a FDP is less meaningful unless it can then be applied to the workplace. Use of projects, in this regard, as an essential element for effective faculty development, is highly recommended [29], and many international FDPs encourage or even instruct participants to conduct projects during or after the program [3, 4, 30]. However, instead of imposing any penalty or formal obligation regarding projects, we constantly emphasized and reminded the fellows about the importance of applying the knowledge and skills they learned throughout the whole course. Moreover, the second design principle emphasized the adaptability of the contents in resource-limited settings. Thus, 3 months after the SICME, there were ongoing or planned projects in every participating country, and this could be a positive sign for improvement in level 3, transfer.

Looking at Fig. 2c and Table 5 together, it appears that the fellows generally had applied module topics they thought were feasible rather than applying module topics that were the urgent or having greater impact. When interpreting this result, we should consider the inherent nature of the following modules—Student Selection and Admissions (Module 6), Accreditation and Licensing Examination (module 7), and Official Development Assistance for Human Resources Development (module 8)—because these are some things that cannot be readily changed simply by individual effort or improved in the short term. It is reasonable to say that the fellows accurately comprehended what could be applied immediately in their current situation. Nevertheless, it is still worth mentioning that feasibility is the key factor in transferring and applying learning in the settings of developing country, at least in the short term.

In addition to the four design principles, multi-level collaboration was another core strategy to overcome challenges that we are likely to face when attempting an international FDP, such as the SICME. First, there was an intraorganizational collaboration inside the host institution. Institutions that took initiative in the SICME have a distinct experience in educational development, either as a donor or as a recipient. In the 1950s, after the Korean War, there was tremendous financial and educational development aid from the U.S. provided to SNUMC in the form of the so-called Seoul National University Cooperative Project [31]. A few decades later, the NTTC was established under the support of the WHO and the China Medical Board in 1975 when South Korea was an underdeveloped country, and it has been running various FDPs since that time [31, 32]. The CGM, established in 2012, as the Regional Education Development Center (REDC) of the Western Pacific Regional Office of the WHO, is actively expanding its central role in educational development in this region. In this developmental process of medical education that has taken place for decades, it is fortunate for the SICME that SNUMC, as South Korea’s representative medical school, is right in the center of change. Particularly, some faculty members of SNUMC who have experienced the change directly have joined the SICME as members of the Planning Committee. Because past experiences of South Korea share many common features with the current situation in developing countries, the past personal experiences of senior members helped the younger members of the committee to significantly improve understanding the actual needs as well as feelings of aid-recipients. This was important to enhance the effectiveness of the SICME because it is not unusual for donors from outside, those who are not familiar with context and operations of the insiders, to face difficulties when trying to address the recipients’ needs [33].

Second, two types of intranational collaboration were key to organizing an English workshop with facilitators and fellows only from non-English-speaking countries. One form of the intranational collaboration was that of the facilitators. For most of the facilitators, who had experienced merely short-term training in a foreign country at most, preparing materials and running workshops in English was no small burden. We did, however, successfully lessen the burden of each facilitator by distributing that burden to more than 20 facilitators from 15 institutions while the module coordinators safeguarded the whole program keeping it aligned with the four design principles. The other was the collaboration among the fellows. The fellows were quite heterogeneous in terms of their English fluency, and sometimes, this was a barrier to delivering important concepts. To solve this problem, we encouraged active peer-tutoring and extensive discussions in their own language whenever they needed. The importance of this interaction is that our intended goal of collaboration among the fellows was not only a means to foster the learning of the educational contents during the SICME, but also a foundation for forming a ‘critical mass’ of leaders in medical education at their own institutions, and in their own regions beyond.

The last one is characterized as continuous international collaboration which encompasses the whole process of the SICME—before, during, and after the program. Before the organization of the SICME, SNUMC and the partner institutions at the five participant countries already have been maintaining cooperative relationships. These relationships generated two main advantages. First, this allowed us to discern and select the most suitable fellows possessing an appropriate background, commitment to learning, and actual leadership to reach the long-term goals of the SICME. Second, it enabled us to develop the SICME program based on the up-to-date information of the participants which enabled us to address their needs more accurately. The positive impact created by the international collaboration among the participant countries did not end with the program. After completion of the program the fellows, rather than SNUMC, actively sought ways for further collaboration. They not only tried to transfer and share their learning through their own efforts, but also voluntarily looked for ways to utilize newly established international relationships by requesting the participation of SNUMC in their projects as an external expert group. There are two reasons that make this especially encouraging. First, projects initiated and led by the recipient side are likely to be more effective than donor-led projects in that they can target the needs and interest of the recipients accurately. Second, from the donors’ side, the fact that a project is proposed by a recipient institution that calls for external support would more strongly justify the necessity of the project and make it easier to prepare budgets for aid.

The evaluation results for levels 1, 2, and 3 showed that we successfully overcame the major inherent challenges of this program through elaborated collaboration. However, there are still some limitations that need consideration. First, our strategy for inviting fellows to South Korea was effective in achieving high satisfaction and active participation in the program, but it places financial restrictions on any major increase in the number of fellows in the following years. More importantly, considering the current positions of the fellows at their institutions, leaving their positions for more than a month or two at a time is also demanding on them. Consequentially, it limits the length, timing, and frequency of the FDP. Ultimately, these limitations might restrict the formation of sufficient’critical masses’ in recipient institutions. Continuing the education of subsequent batches of fellows is important not only because most of the fellows in this study asked for it but also because one-time, short-training courses are at risk of having little or no effect without any further supports [34]. Second, although our data show high levels of satisfaction and significant improvements in learning, previous studies on programs for developing countries pointed out that there is a possibility of overestimating satisfaction or effectiveness of such programs due to fellows’ excessive courtesy or efforts to demonstrate socially desirable attitudes [35]. Third, among the four levels of Kirkpatrick’s model, we only evaluated levels 1, 2, and 3. There are some literature reports about program evaluations that focused only on levels 1, 2, and 3 of Kirkpatrick’s model [35–37], and some researchers have criticized level 4 evaluations for their unrealistic nature for most programs [38]. However tracking of long-term changes in recipient countries is essential, and this needs to be done going forward. Finally, despite our findings that showed that transfer of learning was somewhat connected with the feasibility of the application, further research has to be done to investigate the factors that contribute to and or interrupt the transfer (level 3) and the contexts that ultimately lead to the transfer of learning in a particular country.

## Conclusions

In this study, we implemented a FDP with the aim to help overcome problems in Asian developing countries, especially non-English-speaking countries, and those who are marginalized and only have had scarce opportunities in the area of faculty development. According to the evaluation results, fellows were highly satisfied with the program, and their learning significantly improved after every module. Moreover, there was active transfer of learning, mainly with topics that were highly feasible, depending on their current situation. Even though the format, content, and evaluations have limitations, the overall direction of the observed changes was in line with the initial purpose of the SICME. The foremost task in overcoming the current limitations is supporting current fellows by providing timely feedback at the individual level and establishing cooperative relationships for continuous support at the organizational level in the long term. Future research needs to examine the underlying factors of transfer in various contextual aspects, such as organizational structure, culture, and leadership. These could contribute to further improving the effectiveness of FDPs for developing countries.
